# Definitive host influences the proteomic profile of excretory/secretory products of the trematode *Echinostoma caproni*

**DOI:** 10.1186/s13071-016-1465-x

**Published:** 2016-03-31

**Authors:** Alba Cortés, Javier Sotillo, Carla Muñoz-Antolí, María Trelis, J. Guillermo Esteban, Rafael Toledo

**Affiliations:** Departamento de Parasitología, Facultad de Farmacia, Universidad de Valencia, Av. Vicente Andrés Estellés s/n, 46100 Burjassot, Valencia Spain; Centre for Biodiscovery and Molecular Development of Therapeutics, Australian Institute of Tropical Health and Medicine, James Cook University, Cairns, QLD Australia

**Keywords:** *Echinostoma caproni*, Helminth, Excretory/secretory products, Proteome plasticity, 2-dimensional gel electrophoresis

## Abstract

**Background:**

*Echinostoma caproni* is an intestinal trematode extensively used as experimental model for the study of factors that determine the course of intestinal helminth infections, since this markedly depends on the host species. Although the host-dependent mechanisms for either chronic establishment or early parasite rejection have been broadly studied, little is known regarding the parasite response against different host environments.

**Methods:**

To identify host-dependent differentially expressed proteins, a comparative proteomic analysis of the excretory/secretory products released from *E. caproni* adults, isolated from hosts displaying different compatibility with this trematode, was performed.

**Results:**

A total of 19 differential protein spots were identified (14 overexpressed in mice and 5 overexpressed in rats). The establishment of chronic infections in mice is mainly associated with the overexpression by adult worms of antioxidant and detoxifying enzymes (e.g. glutathione S-transferase, hydroxyacylglutathione hydrolase, thiopurine S-transferase, etc.) and metabolic enzymes like enolase, leucine aminopeptidase or malate dehydrogenase. However, the overexpression of cathepsin L and the structural protein actin observed in worms isolated from rats seems not to be effective for the colonization of the intestinal mucosa of this host.

**Conclusions:**

The observed differences suggest that protein expression and/or release is modulated by the local environment generated inside the host and provide useful insights in regards to the resistance mechanisms developed by parasites to ensure their long-term survival.

**Electronic supplementary material:**

The online version of this article (doi:10.1186/s13071-016-1465-x) contains supplementary material, which is available to authorized users.

## Background

Although intestinal helminth infections are highly prevalent around the world, they are still amongst the most neglected tropical diseases [1] causing a significant impact on the essential components that comprise human development indices [2]. Moreover, parasitic helminth infections in livestock are responsible for significant economic losses in this sector, due to decreases in productivity and the cost of antihelminthic treatments [1]. Recent studies have estimated that around 40 million people are currently infected with food-borne trematodes, including members of the family Echinostomatidae, mainly in the east and southeast Asia [3]. About 20 species belonging to 9 genera of the Echinostomatidae are known to cause human infections worldwide [4, 5], constituting an important group of food-borne trematodes of public health relevance, with prevalence that reaches up to 3 % in some regions of Asia [6, 7].

In addition to their importance for human health, echinostomes, and particularly the species *Echinostoma caproni,* have been used for decades as experimental models to study the relationships between food-borne trematodes and their vertebrate hosts [8, 9]. *Echinostoma caproni* is an intestinal trematode with no tissue phases in the definitive host. After infection, metacercariae excyst in the duodenum and juvenile worms migrate to the ileum, where they attach to the mucosa [9]. This species has a wide range of definitive hosts, although its compatibility markedly differs among rodent species in terms of worm survival and development. In high-compatible hosts, such as mice, the infection is characterized by high worm establishment, high egg output and long-term survival of worms [10]. Rats, conversely, are low-compatible hosts in which worms are expelled from the 2 weeks post-infection (wpi) and worm establishment and egg release are significantly lower than in mice [11]. Other host-dependent phenotypic differences have been reported. Morphological parameters such as body area, collar width or ventral sucker area, amongst others, are larger in high-compatible hosts than in low-compatible ones [10, 11], which has been related to the energy cost required for the greater replacement of tegumental spines that occurs in hosts of low compatibility [12].

Differences in host-parasite compatibility have been mainly attributed to the differential immune response generated by the host against the infection. Previous studies have shown that the establishment of chronic infections in CD1 mice is dependent upon the local production of INF-γ, whereas the early rejection of *E. caproni* in rats is associated with the development of a local Th2/Th17 phenotype with elevated levels of IL-13, IL-17A and IL-23 [13, 14]. Moreover, differential proteomic analyses of the infection-induced intestinal alterations suggest that the expulsion of *E. caproni* in rats is associated with an increased regenerative capacity of the epithelium, mediated by local IL-13 [15]. In contrast, the establishment of chronic infections in mice causes mitochondrial dysfunction in the intestinal epithelial cells and a dysregulation between proliferation and cell death, eventually leading to tissue hyperplasia [16, 17].

Although comparative immunological and pathophysiological studies regarding how hosts enable chronic infections or rapidly promote parasite rejection are extensive in the *E. caproni*-rodent model, there is limited understanding of worm response against different host environments at the proteomic level. In this sense, the study of excretory/secretory and tegumental molecules has led to the discovery of potential candidates for diagnosis, treatment and vaccination against helminthiases [18]. Although the excretory/secretory proteome of *E. caproni* has been previously analyzed [19], herein we followed a new approach based on comparative proteomics. Host-dependent differentially expressed proteins are studied in the excretory/secretory products (ESPs) of *E. caproni* adult worms obtained from experimentally infected mice and rats to investigate proteome plasticity and identify proteins involved in parasite adaptation that enable its long-term establishment in the host. Quantitative differences were evaluated by single stained 2-dimensional gel electrophoresis (2DGE), using Progenesis SameSpots software. This experimental approach has been proved a consistent and reliable method for the detection and matching of protein spots from other pathogenic organisms [20, 21].

## Methods

### Parasites and experimental infections

The strain of *E. caproni* and the infection procedures have been previously described [22]. Briefly, encysted metacercariae of *E. caproni* were removed from kidneys and pericardial cavities of experimentally infected *Biomphalaria glabrata* snails and used to infect CD1 mice and albino Wistar rats. A total of 9 mice (male, 5 week-old) and 9 rats (male, 3 week-old) were infected by gastric gavage with 75 and 100 metacercariae, respectively. All animals were necropsied at 4 weeks post-infection (wpi) and adult parasites were used to obtain ESPs. The animals were maintained under standard conditions with food and water *ad libitum*. This study has been approved by the Ethical Committee of Animal Welfare and Experimentation of the University of Valencia (Ref#A18348501775). Protocols adhered to Spanish (Real Decreto 53/2013) and European (2010/63/UE) regulations.

### Isolation of ESPs

**Fig. 1:**
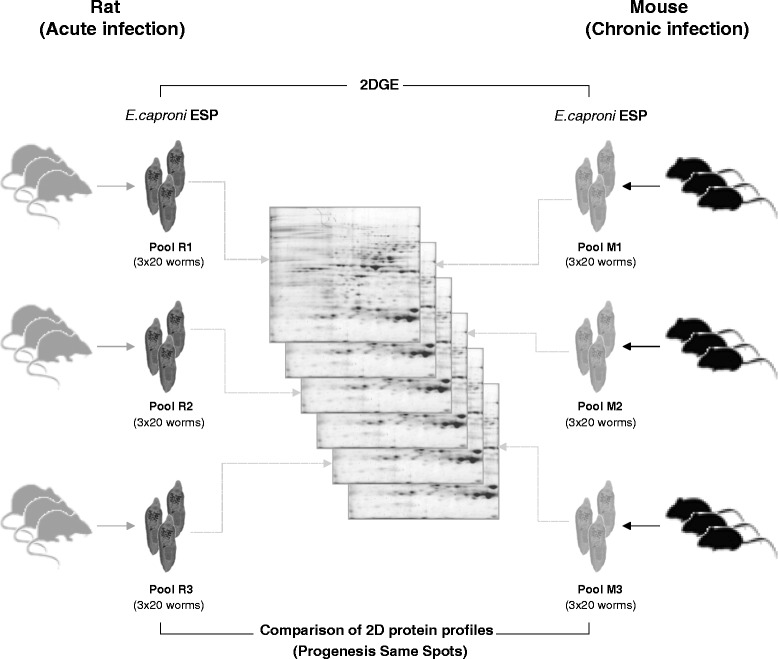
Experimental strategy for the comparison of the excretory/secretory proteomes of *Echinostoma caproni* adult worms isolated from mice and rats

To recover the ESPs of *E. caproni*, adult worms were collected from the intestine of experimentally infected mice and rats at 4 wpi. After collection, parasites were washed with pre-heated RMPI culture medium (Gibco®, Life Technologies) and maintained at a concentration of 10 worms/ml for 12 h, at 37 °C, in RPMI containing cOmplete mini EDTA-free protease inhibitor cocktail (Roche). The medium was then collected and centrifuged at 15,000 *g* for 30 min at 4 °C. After centrifugation, the supernatant was collected and protein concentration was measured using Bio-Rad protein assay. To increase the biological variability of samples, a total of 3 biological replicates were employed. Each replicate was prepared by incubating a total of 60 worms recovered from 3 different mice or rats (20 worms from each host). The experimental design is outlined in Fig. 1.

### Analysis of ESP proteins by 2-Dimensional Gel Electrophoresis (2DGE)

The 2DGE was carried out essentially as previously described [23], solubilizing protein samples in 7 M urea, 2 M thiourea, 4 % CHAPS (w/v), 20 mM DTT and 2 % (v/v) Biolytes 3–10, containing bromophenol blue (all chemicals from Bio-Rad). Loads of 150 μg of protein were applied onto linear pH 5-8 IPG gels (7 cm length, Bio-Rad) and subjected to isoelectric focusing on a Bio-Rad PROTEAN® IEC Cell at 20 °C using the following program: (i) passive rehydration for 16 h; (ii) step 300 V for 1 h; (iii) gradient 4,000 V for 2 h and (iv) step 4,000 V for 6.5 h. After the electric focusing, the strips were reduced (2 % DTT) and then alkylated (2.5 % iodoacetamide) in equilibration buffer containing 6 M urea, 0.375 Tris pH 8.8, 2 % SDS and 20 % glycerol. The second dimension was performed using 12 % polyacrylamide gels with Tris-glycine SDS buffer. The resolved proteins were detected using Silver Stain Plus kit (Bio-Rad).

### Comparison of 2D protein profiles

Quantitative proteomic analysis of the ESPs of *E. caproni* adults isolated from high- and low-compatible hosts was carried out using Progenesis SameSpots software (version 4.5) (Nonlinear Dynamics Ltd.). The comparison of single-stained 2DGE data was performed as previously described by Smith *et al.* [20] with slight modifications. Triplicate 2D gels of the ESPs of *E. caproni* adults from mice and rats were analyzed. Each sample was prepared from a pool of parasites recovered from different infected animals, hence representing three independent biological replicates (Fig. 1).

Dried, silver-stained 2D gels were scanned using an ImageScaner III (GE Helthcare) to generate 16-bit grey level images at resolution of 600 dpi. Firstly, gel images were normalized to minimize experimental variation and allow multiple comparisons. After normalization, 2D profiles were aligned using a combination of manually and automatically generated vectors. Spot detection was performed with the in-built software routines and the outlines transferred across the whole of the gel series, and it was manually supervised to ensure that each spot was well defined. Minimal editing was needed to exclude artifacts, overlapping spots or single spots that were recognized as two. The edited datasets were transferred to the Progenesis SameSpots statistical package for analysis. Protein spots showing statistically significant differences in abundance between the ESPs of parasites recovered from mice and rats were selected using Student’s *t*-test (*P* < 0.01) and False Discovery Rate (FDR) (*q* < 0.05).

### Protein identification by mass spectrometry (MS) and database search

Those spots showing significant host-dependent differences were manually excised from gels, washed twice with double-distilled water and digested with sequencing grade trypsin (Promega). Digested samples were diluted in 12 μl of 5 % formic acid and 6 μl of the resulting suspension were injected onto a 50 mm × 300 μm C18 trap column (Agilent Technologies) using a Shimadzu Prominance Nano HPLC. Samples were desalted on the trap column for 5 min using 0.1 % formic acid (aq) at 30 μl/min. Peptides were then eluted onto an analytical nano HPLC column (150 mm x 75 μm 300SBC18, 3.5 μm, Agilent Technologies) at a flow rate of 300 nL/min and separated using a 35 min gradient of 1–40 % buffer B followed by a steeper gradient from 40–80 % buffer B in 5 min. Buffer B contained 90/10 acetonitrile/0.1 % formic acid, and buffer A consisted of 0.1 % formic acid (aq). The column eluates were subsequently ionized using a 5500 QTRAP system (AB Sciex) operated in an Information Dependent Acquisition, IDA, mode. Full scan TOFMS data was acquired over the mass range 350–1400, and for product ion MS/MS 80–1400 m/z ions observed in the TOF-MS scan exceeding a threshold of 100 counts and a charge state of +2 to +5 were set to trigger the acquisition of product ion, MS/MS spectra of the resultant 20 most intense ions.

Database search was performed using X!Tandem and MS-GF+ search engines on the *E. caproni* genome database, available on-line at http://parasite.wormbase.org/Echinostoma_caproni_prjeb1207/Info/Index/, and on the *E. caproni* transcriptome database [24]. Searches were done with tryptic specificity, allowing two missed cleavage and a tolerance in mass measurement of 10 ppm in MS mode and 0.5 Da for MS/MS ions. Carbamidomethylation of Cys was used as fixed modification and oxidation of Met and deamidation of Asn and Gln as variable modifications. Only proteins identified with 2 or more validated peptides were taken into account. Search results were imported into PeptideShaker v.1.2.2 [25] for peptide and protein inference. Only proteins with a false dscovery rate < 1 %, having at least two unique peptides (containing at least seven amino acid residues) were considered as positively identified. Proteins were classified according to Gene Ontology (GO) categories using the software Blast2GO basic version 3.1 [26].

### Validation by 1DGE and western blotting

In order to validate the usage of Progenesis SameSpots software for the comparison of the ESP proteomes of *E. caproni*, western blot analyses were performed on several differential proteins for which either homologous or heterologous antibodies were available. For the 1DEG, 40 μg of protein were loaded onto each lane of 10 % resolving and 4 % stacking polyacrylamide gels and electrophoresed in Tris-glycine SDS buffer. Proteins were transferred to nitrocellulose membranes (0.45 mm) in 20 mM Tris, 192 mM glycine and methanol 20 %, pH 8.3. After 90 min blocking with 5 % skimmed milk in phosphate saline buffer containing 0.05 % of Tween-20 (PBS-T), blots were incubated for 2 h in PSB-T with the following antisera: rabbit polyclonal anti-*E. caproni* enolase (1/2,000) [23]; rabbit polyclonal anti-*E. caproni* actin (1/2,000) [27]; rabbit polyclonal anti-*Fasciola hepatica* leucine aminopeptidase (LAP; 1/4,000), kindly provided by Dr. C. Carmona, Universidad de la República, Montevideo, Uruguay [28]; and sheep polyclonal anti-*F. hepatica* cathepsin L1 (1/1,000), kindly provided by professor J. P. Dalton, Queen’s University, Belfast, United Kingdom [29]. Membranes were then washed and probed with peroxidase-conjugate secondary antibodies: goat anti-rabbit IgG (1/10,000 for enolase; 1/8,000 for actin and 1/16,000 for LAP) and rabbit anti-sheep IgG (1/5,000 for cathepsin L1) in PBS-T for 2 h. Negative controls were performed by incubating the same amount of protein with sera from hosts, i.e. rabbit or sheep, immunized with PBS. All the incubations were performed at room temperature and under gentle agitation. Blots were developed with Lumi-Light Western Blotting Substrate (Roche) following the manufacturer’s instructions and images were taken with a ChemiDoc™ imaging system (Bio-Rad). The bands were quantified using the image analysis software ImageJ (National Institutes of Health).

## Results

### *Comparison of host-dependent* E. caproni-*ESP proteomes*

**Fig. 2:**
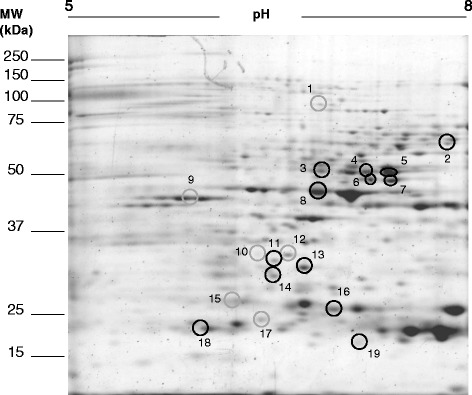
Representative 2-dimensional gel electrophoresis image. Grey circles indicate spots overexpressed in the excretory/secretory products (ESPs) of rats and black circles indicate spots overexpressed in the ESPs of mice

**Fig. 3:**
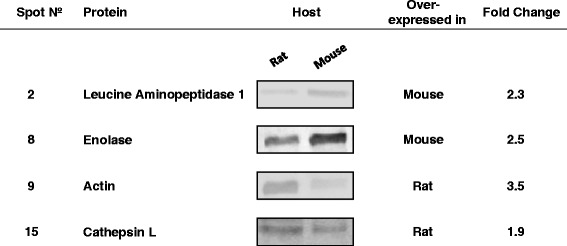
Western blot validation of several differentially expressed protein spots. Representative images of blots confirming host-dependent differential expression of the four tested proteins. Validations were performed in duplicate, employing distinct biological replicates from each host. Fold changes were calculated using ImageJ software (National Institutes of Health) on the two replicates, and are presented as the ratio between the mean values calculated for each host species. Spot numbers refer to Fig. 2 and Table 1

In order to identify host-dependent differentially expressed proteins, ESPs of *E. caproni* adults obtained from mice and rats were subjected to 2DGE and 2D-gel images were analyzed with Progenesis SameSpots software (Fig. 1). A total of 883 protein spots matched through the 6 gels included in the analysis, and 56 of them showed significant statistical differences between the two groups (*P* < 0.01 and *q* < 0.05). Thirty protein spots were overexpressed in the ESPs of worms recovered from mice (hereinafter, overexpressed in mice), whereas the reminder 26 showed a greater expression in the ESPs of *E. caproni* adults isolated from the intestine of rats (hereon, overexpressed in rats). Finally, 19 of these differential spots were successfully identified by MS and database search: 14 overexpressed in mice and 5 overexpressed in rats (Fig. 2 and Additional file 1). Fold differences (F) between the average normalized volumes for each host species ranged between 1.5 and 5.3, and the details of the computational comparison can be seen in Additional file 2. The validity of this comparison was assessed by 1DGE and western blot using polyclonal antibodies against 4 proteins (LAP, enolase, cathepsin L and actin), that were overexpressed either in mice or in rats (Fig. 3). Host-dependent differential expression was confirmed for the 4 proteins, thus strengthen the reliability of inter-gel matches and gel-based protein quantification.

### Identification of host-dependent differentially expressed ESP proteins

To avoid erroneous identifications due to mismatched spots, protein identification was performed in duplicate. Selected protein spots were removed from a gel of mouse and a gel of rat, and were independently identified by MS. Only when the two identifications coincided the protein was considered to be unambiguously identified. Moreover, database search was performed employing specific protein sets based on both *E. caproni* transcriptome [24] and genome assemblies. Details of protein identification in each database are shown in Additional file 3. A total of 19 protein spots were identified using the transcriptome database. However, neither dihydrolipoamide dehydrogenase, nor malate dehydrogenase were identified when genome data was used (Table 1 and Additional file 3).

**Table 1:** Identification of differentially expressed proteins

Spot^a^	Identification^b^		Protein^c^	Species (GI)^d^	Host^e^	Fold Change^f^	MW Exp/Theo	pI Exp/Theo
	Genome	Transcriptome						
Antioxidant and detoxifying enzymes								
6		√	Dihydrolipoamide dehydrogenase	*Schistosoma japonicum* (226486712)	M	5.3	47.8/53.0	7.2/6.5
10	√	√	Aldo-keto reductase	*S. mansoni* (256080704)	R	2.9	33.6/33.5	6.4/7.7
12	√	√	Aldo-keto reductase	*S. mansoni* (256080704)	R	4.6	33.0/33.5	6.6/7.7
13	√	√	Aldo-keto reductase	*S. mansoni* (350645579)	M	2.0	31.3/35.5	6.7/7.7
16	√	√	Hydroxyacylglutathione hydrolase	*Clonorchis sinensis* (358335388)	M	1.8	25.0/29.6	6.9/6.9
17	√	√	Thiopurine S-methyltransferase	*S. japonicum* (226484786)	M	2.1	23.0/28.1	6.7/7.1
19	√	√	Glutathione S-transferase class-mu	*Fasciola hepatica* (3913799)	M	1.8	16.5/25.3	7.1/5.9
Metabolic enzymes								
2	√	√	Leucine aminopeptidase	*C. sinensis* (156600435)	M	1.5	61.5/59.9	7.8/6.1
3	√	√	Retinal dehydrogenase 1	*C. sinensis* (358342257)	M	3.5	50.0/52.6	6.8/6.0
4	√	√	Hexokinase	*C. sinensis* (358342257)	M	1.9	48.7/50.0	7.2/6.2
5	√	√	6-phosphogluconate dehydrogenase	*S. haematobium* (844860554)	M	2.2	48.7/52.8	7.3/6.2
7		√	Malate dehydrogenase	*C. sinensis* (358332642)	M	5.1	47.8/55.7	7.3/6.6
8	√	√	Enolase	*Echinostoma caproni* (112950027)	M	1.7	45.5/46.1	6.8/6.4
Structural/Motor proteins								
9	√	√	Putative actin	*S. mansoni* (353233111)	R	2.0	43.7/41.7	5.9/5.3
18	√	√	Regulator of microtubule dynamics protein 1	*S. haematobium* (844834702)	M	1.9	21.0/24.3	6.0/8.6
Cysteine proteases								
15	√	√	Cathepsin L	*F. gigantica* (7271891)	R	2.3	26.1/37.0	6.1/5.5
Protein binding								
11	√	√	Stress-induced phosphoprotein 1	*C. sinensis* (350002666)	M	2.8	32.4/36.3	6.5/6.3
Unknown								
1^g^	√	√	Periostin	*C. sinensis* (358341487)	R	2.9	90.0/105.3	6.8/5.8
	√	√	Fasciclin 1-like	*Paragonimus westermani* (119712173)				
	√	√	Gynecophoral canal protein	*S. mansoni* (1354127)				
14	√	√	Putative TyrA protein	*S. japonicum* (226479962)	M	2.8	32.4/41.6	6.5/7.0

Protein identification was performed using X!Tandem and MS-GF+ search engines on the *Echinostoma caproni* genome and transcriptome databases and BLASTp against NCBInr protein database. Spot numbers refer to gel image in Fig. 2

^a^Spot reference number

^b^Positive identification (√) in each of the two databases employed

^c^Identification details are compiled in Additional file 3

^d^GI accession number in the Protein database of NCBI

^e^Host species in which every protein spot was overexpress. (M) Mouse; (R) Rat

^f^Average Normalized Volume ratio

^g^Three different proteins were associated with the same accession number both in the *E. caproni* genome and transcriptome databases

In order to investigate the processes and functions overrepresented in each ESP, proteins were classified according to GO categories. This classification was not possible in the case of rats due to the small number of identified proteins. In mice, however, proteins with enzymatic activity and involved both in energy and non-energy metabolism were highly abundant (Additional files 4 and 5). Among the enzymes involved in energy metabolism there were the glycolytic enzymes hexokinase (F: 1.9) and enolase (F: 1.7); malate dehydrogenase (F: 5.1), which is involved in the Krebs cycle; and 6-phosphogluconate dehydrogenase (F: 2.2), an enzyme in the pentose phosphate pathway. LAP (F: 1.5) and retinal dehydrogenase (F: 3.5) are metabolic enzymes involved in non-energy metabolism that were also overexpressed in mice (Table 1).

The protein group displaying a greater number of differentially expressed spots was that of antioxidant and detoxifying enzymes. In this group, 3 differential spots [2 overexpressed in rats (F: 4.6 and 2.9) and another one overexpressed in mice (F: 2.0)] were identified as aldo-keto reductases. Other antioxidant and detoxifying enzymes such as hydroxyacylglutathione hydrolase (F: 1.8), dihydrolipoamide dehydrogenase (F: 5.3) and glutathione S-transferase (GST, F: 1.8) were overexpressed in mice (Table 1). Among proteins overexpressed in rat we found the structural protein actin (F: 2.0), cathepsin L (F: 2.3) and a protein with fasciclin 1-like domains, which could not have been accurately identified (spot number 1, F: 2.9).

## Discussion

ESPs are composed of a complex mixture of molecules, including proteins, lipids, nucleic acids, etc. Protein secretion is known to occur through different ways, including: 1) classical, signal peptide-driven secretion; 2) non-classical secretion; and 3) through secretory vesicles like exosomes. Independently of the way for release, every protein present in the extracellular milieu is susceptible of playing a role at the host-parasite interface, as they are crucial for the interaction between parasites and their hosts during infection and pathology. Moreover, it has been demonstrated that not only parasite-derived proteins can modulate the host immune response [30], but also changes in the intestinal cytokine milieu directly influence the parasite proteome and this may affect its establishment [31, 32]. The *E. caproni*-rodent model is a well-established experimental model for the analysis of interactions between adult parasites and their vertebrate hosts [8]. A number of studies have been performed and showed that host-related factors are determining for the course of the infection [9, 12–17]. However, little is known about how each environment affects the phenotype of the parasite and its potential implications in terms of worm rejection or chronic establishment.

In this study, host-dependent ESP profiles are investigated using a quantitative 2D-proteomic approach. By combining the available 'omic data on *E. caproni* (i.e. genome and transcriptome assemblies), we aimed at increasing the number and accuracy of our identifications. Sequences for dihydrolipoamide dehydrogenase (spot 6) and malate dehydrogenase (spot 7) were not found in the genome assembly of *E. caproni* when homologous sequences from other trematodes were used for blasting in this database. This suggests that these genes may have been mis-annotated, and reinforce the idea that the combination of several databases may aid to increase the number of protein identifications in the case of non-model organisms. However, despite the combination of DNA- and RNA-based data, an accurate identification of the protein of interest is not always possible due to the lack of specific proteomic data [33]. This has been the case of the differential spot number 1 (Fig. 1). Both genome- and transcriptome-derivative sequences display significant similarity with 3 different proteins from parasitic trematodes belonging to distinct genera (Table 1 and Additional file 3). Although these 3 proteins have the common feature of containing fasciclin 1-like domains, each one has different functions, making difficult to infer its role in the interaction with the host in the case of *E. caproni* infections.

Several enzymes involved both in energy and non-energy metabolism were highly abundant among the proteins overexpressed in mice. Although these are typically intracellular proteins, they are usually present extracellularly, either secreted or attached to parasite surface, where they can carry out atypical functions and participate in host-parasite interactions [34]. Enolase is one of the most commonly found extracellular metabolic enzymes and has been described as the most antigenic protein in the ESPs of *E. caproni* [23]. Furthermore, *E. caproni* enolase has been suggested of great importance for the attachment to the host intestinal mucosa, due to its ability to bind plasminogen *in vitro* [35]. More recently, the enolase of the protozoan parasite *Leishmania donovani* has been demonstrated to be Th1-stimulatory, with the recombinant protein displaying a strong ability to proliferate lymphocytes along with significant IL-12, IFN-γ and nitric oxide production [36]. The over-secretion of this enzyme in adults obtained from mice, which develop strong Th1 local responses, may indicate therefore the immunomodulatory ability of the *E.caproni* enolase, favoring the development of type 1 responses that are important both for the chronic establishment of the parasite [14] and the protection of chronically infected high-compatible hosts [37].

Similarly, LAP is a cytosolic metalloprotease involved in digestion and, possibly, invasion and migration through the host tissues [38], being a potential drug target and vaccine candidate against helminthiases [39, 40]. The overexpression of this enzyme in the high-compatible host suggests that it may have a role in the colonization of the intestinal mucosa by *E. caproni*.

Antioxidant and detoxifying enzymes were predominant among the identified proteins. Three differential spots, 2 of them overexpressed in rats and the other one overexpressed in mice, were identified as aldo-keto reductases (Table 1), a superfamily of NAD(P)(H) oxidoreductases involved in the reduction of aldehydes and ketones to primary and secondary alcohols. The overexpression of different spots depending on the host species may indicate that the expression of different members of this superfamily is somehow regulated and depends upon the environment inside the host.

Parasites causing long-lasting infections are exposed to high amounts of reactive oxygen and nitrogen species generated as a result of the host immune response, which ultimately lead to parasite death [41]. GST is a key enzyme at this first line of defense, having a major role in limiting the damage caused by nitroxidative stress [41]. Previously localized in the tegument and the ESPs of several trematode species, including *E. caproni* [19, 42, 43], herein GST was found about two times overexpressed in the ESPs of adults obtained from the intestine of mice. The establishment of *E. caproni* chronic infections in mice coincides with the development of local Th1-type responses, with high mRNA expression of IFN-γ and iNOS [14], and the population of mucosal neutrophils rapidly increasing at the site of infection [10]. Hence, in this scenario an intense activation of antioxidant systems seems to be imperative to ensure the long-term survival of the parasite. The expulsion of *E. caproni* from the rat intestine, in contrast, has been associated with the development of local Th2/Th17 biased responses, in the absence of increased IFN-γ nor iNOS mRNA expression [12, 13]. In this host, intense eosinophil infiltration has been reported in the intestinal mucosa [44]. Eosinophil peroxidase is responsible of direct oxidative damage on parasite surface [45]. Thus, according to our results, a lesser expression of GST by *E. caproni* adults in the intestine of rats may increase their susceptibility to the granulocyte-mediated immunity, thereby impairing their establishment in the intestine of rats. Furthermore, hydroxyacylglutathione hydrolase and dihydrolipoamide dehydrogenase are detoxifying enzymes previously described in the ESPs of other parasitic trematodes [46, 47], and their high secretion may contribute to the chronic establishment of the parasite in the high-compatible host.

Instead of self-protective proteins such as antioxidant and detoxifying enzymes, *E. caproni* adults isolated from rats seem to over-release proteins related to worm invasiveness, such as cathepsin L and actin. Cathepsins are papain-like endopeptidases, used by helminth parasites in essential aspects of their relationship with the host [48]. Much available knowledge on cathepsins of infectious helminths comes from tissue dwelling trematodes such as *F. hepatica* or *Schistosoma* spp. [49, 50]. However, non-helminthic intestinal parasites have been also shown to secret cathepsin proteases that are able to cleave the non-glycosylated ends of the soluble mucin Muc2, degrading the polymeric structure of mucus matrix and enabling the invasion of the epithelium [51, 52]. Moreover, protease release is considered a resistance mechanism against mucus-mediated helminth clearance [53]. The over-secretion of cathepsin L in adults obtained from low-compatible hosts is, therefore, surprising and difficult to explain according to our current knowledge. Notwithstanding, mucus hyper-secretion seems not to be involved in the expulsion of *E. caproni* in rats [54]. Overexpression of actin, however, is in agreement with previous studies performed with both *E. caproni* [12] and the related species *Echinostoma friedi* [55], and has been attributed to an accelerated turnover of tegumentary spines with the aim of increase the anchoring capacity to the mucosal surface of non-permissive hosts [12].

## Conclusions

Host species has been shown to be determining for the course of *E. caproni* infections in terms of worm survival and development. However, the parasite response against each particular environment is still poorly understood. In the present paper, we have shown the proteome plasticity of *E. caproni* adult worms through the identification of host-dependent differentially expressed and/or released proteins. Both parasites and hosts are able to influence each other. Our results seem to indicate that, additionally to immune-mediated mechanisms, a poor adaptation of *E. caproni* to the microhabitat generated in the rat intestine may facilitate its rapid rejection from this host. In contrast, the ESP profile of mice-grown worms suggests a better adaptation of the parasite to the Th1-type environment developed in the intestine of this host, thereby enabling its chronic establishment. The identification of precise host-dependent mechanisms that govern parasite adaptation appears as a suitable approach to develop new strategies for the control of helminthic infections. Moreover, the identification of proteins implicated in parasite resistance may help to recognize useful targets for drug and vaccine development.

### Ethics approval and consent

The present study has been approved by the Ethical Committee of Animal Welfare and Experimentation of the University of Valencia (Ref#A18348501775), with protocols adhered to Spanish (Real Decreto 53/2013) and European (2010/63/UE) regulations.

### Availability of data and material

The datasets supporting the conclusions of this article are included within the article and its additional files.

## Additional files

Additional file 1: Detailed gel images of the differential spots identified by mass spectrometry and database search. Magnification of the 2D gel-areas corresponding to the 19 differentially expressed spots identified by mass spectrometry and database search. Detailed images for each of the 6 replicates analyzed (3 corresponding to the ESPs of *E. caproni* adults obtained from rats, and 3 from those isolated from mice) are shown. Spot numbers refer to gel image in Fig. 2. (PPTX 4834 kb) Additional file 2: Details of the computational comparison of the excretory/secretory proteomes. Description of data: Quantitative and statistical details of the computational comparison of excretory/secretory proteomes of *Echinostoma caproni* adult worms isolated from mice and rats using Progenesis SameSpots software (version 4.5) (Nonlinear Dynamics Ltd.). Spot numbers refer to gel image in Fig. 2. (DOCX 13 kb) Additional file 3: Identification details of differentially expressed proteins. Description of data: Details of the identification of differentially expressed proteins using X!Tandem and MS-GF+ search engines on the *Echinostoma caproni* genome and transcriptome databases and BLASTp analysis against NCBInr protein database. Spot numbers refer to gel image in Fig. 2. (DOCX 19 kb) Additional file 4: Biological process-based classification of proteins overexpressed in mice. Significantly overexpressed proteins in the excretory/secretory products of *Echinostoma caproni* adults obtained from mice, classified according to their Gene Ontology (GO)-predicted biological process. Pie chart represents the number of proteins assigned to each GO category (biological process, level 4). Proteins included in each category are listed below the graph. (PPTX 118 kb) Additional file 5: Molecular function-based classification of proteins overexpressed in mice. Significantly overexpressed proteins in the excretory/secretory products of *Echinostoma caproni* adults obtained from mice, classified according to their Gene Ontology (GO)-predicted molecular function. Pie chart represents the number of proteins assigned to each GO category (molecular function, level 3). Proteins included in each category are listed below the graph. (PPTX 77 kb)

## Abbreviations

2DGE: two-dimensional gel electrophoresis
ESPs: excretory/secretory products
F: fold difference
FDR: false discovery rate
GO: gene onthology
GST: glutathione S-transferase
LAP: leucine aminopeptidase
MS: mass spectrometry
PBS-T: phosphate saline buffer with Tween-20 (0.05 %)
Wpi: weeks post-infection
