# Supplementary material for: Definitive host influences the proteomic profile of excretory/secretory products of the trematode Echinostoma caproni
Source: Parasit Vectors. 2016 Mar 31;9:185. doi: 10.1186/s13071-016-1465-x (PMC4815245; doi:10.1186/s13071-016-1465-x)
Supplement: Additional file 1: — Detailed gel images of the differential spots identified by mass spectrometry and database search. Magnification of the 2D gel-areas corresponding to the 19 differentially expressed spots identified by mass spectrometry and database search. Detailed images for each of the 6 replicates analyzed (3 corresponding to the ESPs of E. caproni adults obtained from rats, and 3 from those isolated from mice) are shown. Spot numbers refer to gel image in Fig. 2. (PPTX 4834 kb) [file 13071_2016_1465_MOESM1_ESM.pptx]

## Slide 1
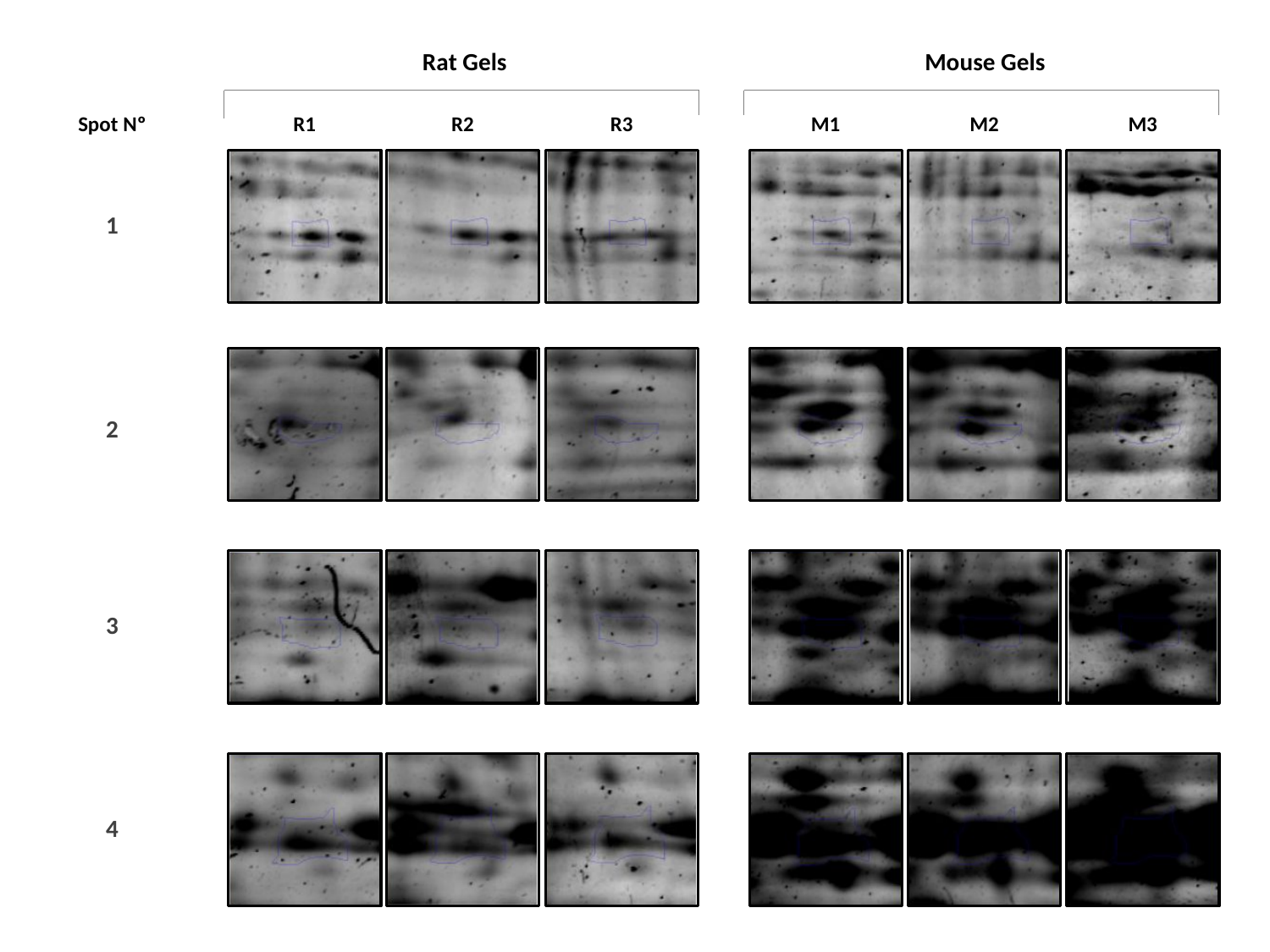

Rat Gels
Mouse Gels
Spot Nº
R1
R2
R3
M1
M2
M3
1
2
3
4

## Slide 2
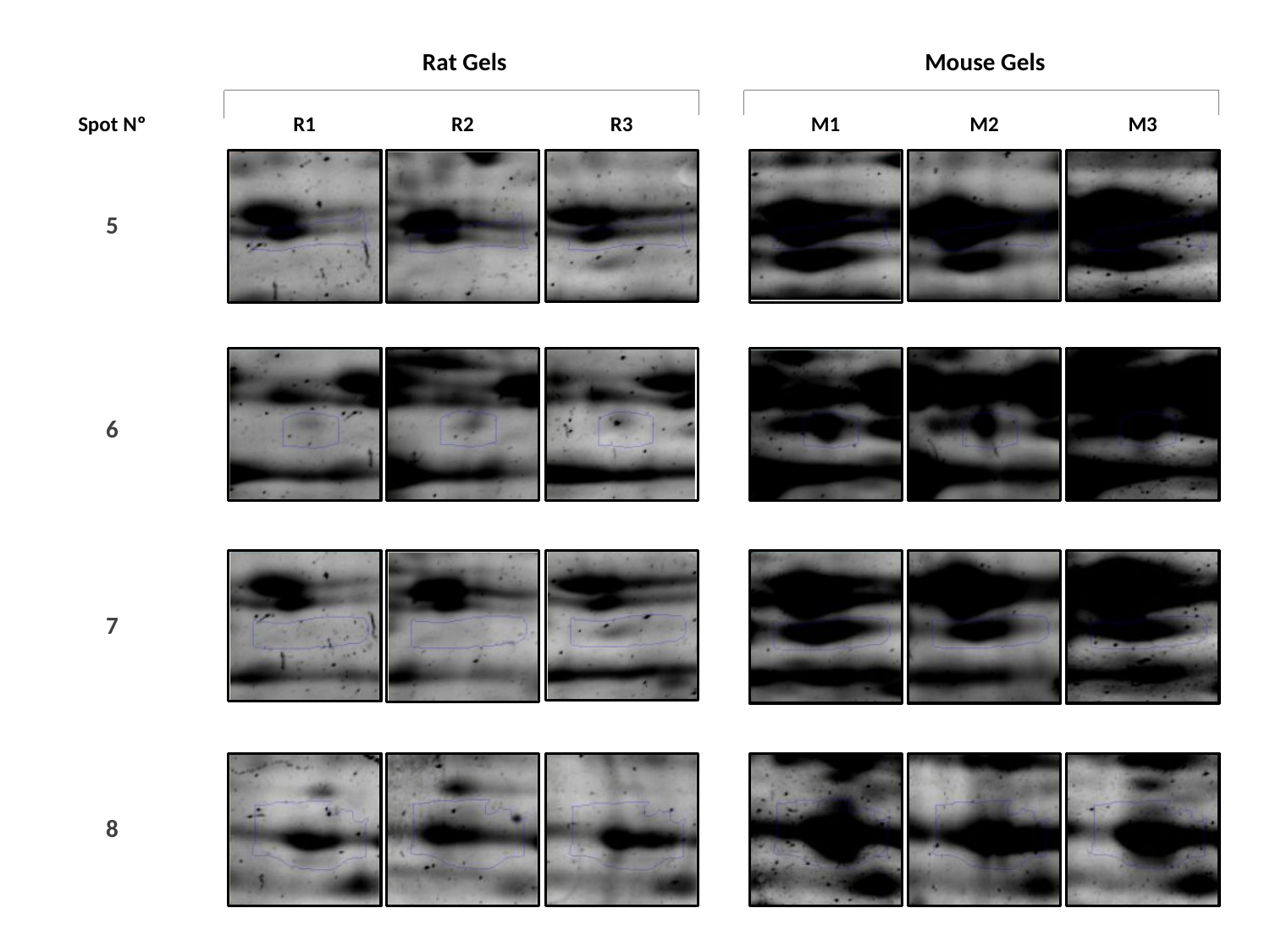

Rat Gels
Mouse Gels
Spot Nº
R1
R2
R3
M1
M2
M3
5
6
7
8

## Slide 3
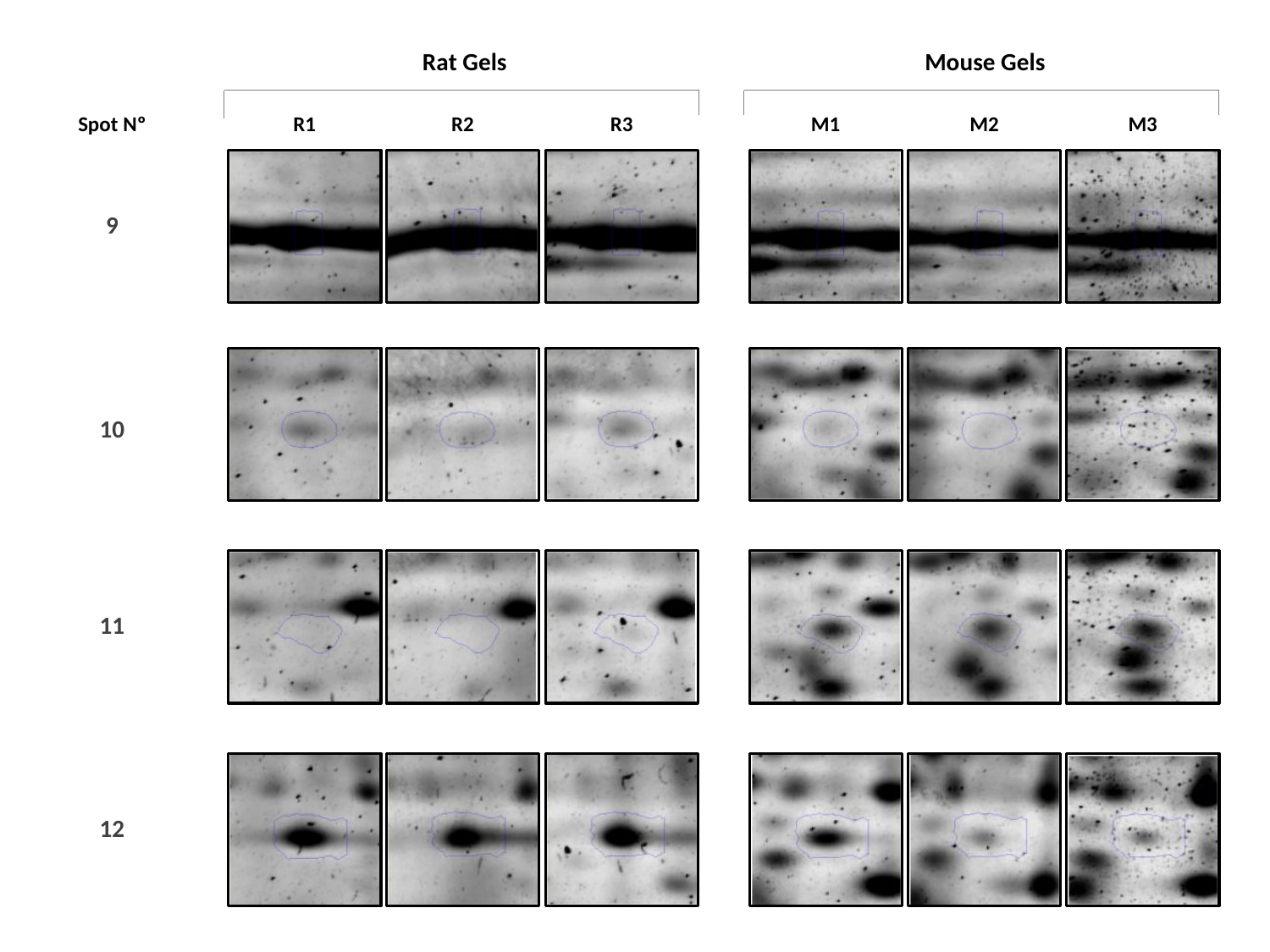

Rat Gels
Mouse Gels
Spot Nº
R1
R2
R3
M1
M2
M3
9
10
11
12

## Slide 4
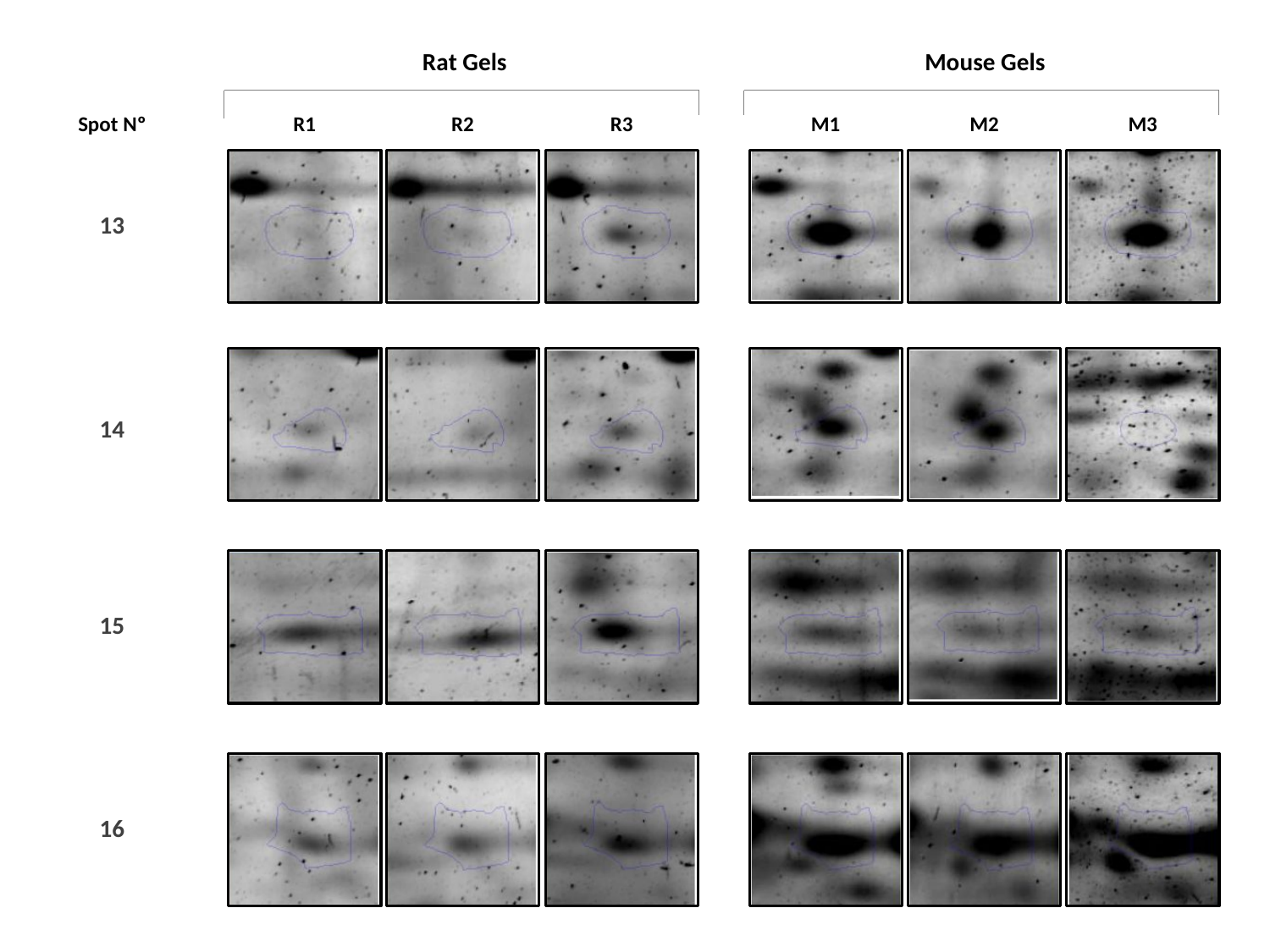

Rat Gels
Mouse Gels
Spot Nº
R1
R2
R3
M1
M2
M3
13
14
15
16

## Slide 5
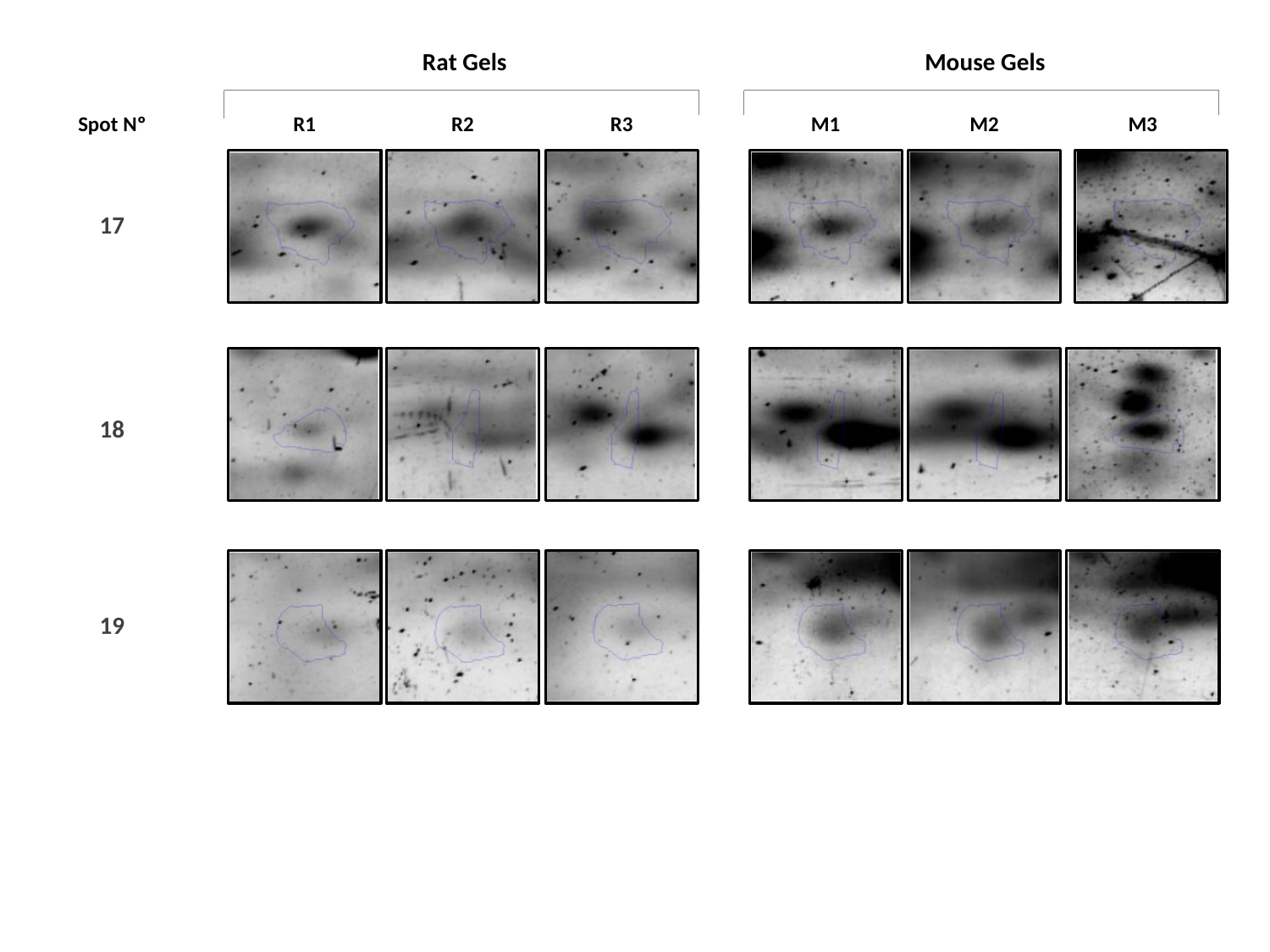

Rat Gels
Mouse Gels
Spot Nº
R1
R2
R3
M1
M2
M3
17
18
19
