# Supplementary material for: Definitive host influences the proteomic profile of excretory/secretory products of the trematode Echinostoma caproni
Source: Parasit Vectors. 2016 Mar 31;9:185. doi: 10.1186/s13071-016-1465-x (PMC4815245; doi:10.1186/s13071-016-1465-x)
Supplement: Additional file 2: — Details of the computational comparison of the excretory/secretory proteomes. Description of data: Quantitative and statistical details of the computational comparison of excretory/secretory proteomes of Echinostoma caproni adult worms isolated from mice and rats using Progenesis SameSpots software (version 4.5) (Nonlinear Dynamics Ltd.). Spot numbers refer to gel image in Fig. 2. (DOCX 13 kb) [file 13071_2016_1465_MOESM2_ESM.docx]

| **Spot^a^** | **AVN^b^ Rat (x10^6^)** | **AVN^b^ Mouse (x10^6^)** | **Overexpressed in** | **Fold change^c^** | ***t* test *p*^d^** |
| --- | --- | --- | --- | --- | --- |
| 1 | 6.57 | 2.30 | Rat | 2.9 | 0.003 |
| 2 | 6.23 | 9.27 | Mouse | 1.5 | 0.002 |
| 3 | 7.80 | 27.22 | Mouse | 3.5 | 0.002 |
| 4 | 30.57 | 57.56 | Mouse | 1.9 | 0.006 |
| 5 | 28.99 | 63.51 | Mouse | 2.2 | 3.47*10^-5^ |
| 6 | 5.13 | 26.92 | Mouse | 5.3 | 0.002 |
| 7 | 9.36 | 47.44 | Mouse | 5.1 | 0.003 |
| 8 | 67.99 | 118.00 | Mouse | 1.7 | 0.002 |
| 9 | 37.82 | 18.47 | Rat | 2.0 | 0.004 |
| 10 | 5.91 | 2.05 | Rat | 2.9 | 1.27*10^-4^ |
| 11 | 3.32 | 9.22 | Mouse | 2.8 | 9.92*10^-5^ |
| 12 | 38.92 | 8.52 | Rat | 4.6 | 0.006 |
| 13 | 16.29 | 32.14 | Mouse | 2.0 | 0.001 |
| 14 | 7.25 | 14.40 | Mouse | 2.0 | 0.005 |
| 15 | 27.56 | 12.03 | Rat | 2.3 | 0.001 |
| 16 | 24.27 | 44.79 | Mouse | 1.8 | 8.87*10^-4^ |
| 17 | 4.08 | 8.39 | Mouse | 2.1 | 0.002 |
| 18 | 7.73 | 14.50 | Mouse | 1.9 | 0.005 |
| 19 | 6.70 | 11.75 | Mouse | 1.8 | 0.007 |

^a^ Spot reference number.

^b^ AVN: Average Normalized Volume.

^c^ AVN ratio.

^d^ *p* value of Student’s *t*-test.
