# Supplementary material for: Definitive host influences the proteomic profile of excretory/secretory products of the trematode Echinostoma caproni
Source: Parasit Vectors. 2016 Mar 31;9:185. doi: 10.1186/s13071-016-1465-x (PMC4815245; doi:10.1186/s13071-016-1465-x)
Supplement: Additional file 3: — Identification details of differentially expressed proteins. Description of data: Details of the identification of differentially expressed proteins using X!Tandem and MS-GF+ search engines on the Echinostoma caproni genome and transcriptome databases and BLASTp analysis against NCBInr protein database. Spot numbers refer to gel image in Fig. 2. (DOCX 19 kb) [file 13071_2016_1465_MOESM3_ESM.docx]

| **Identification details** | | | | | | | **Blastp details^g^** | | | |
| --- | --- | --- | --- | --- | --- | --- | --- | --- | --- | --- |
| **Spot^a^** | **Protein** | **Species**  **(GI)^b^** | **DB^c^** | **Accesssion number^d^** | **Cov.^e^** | **Val. Pept.^f^** | **E-value** | **Total score** | **Query Cov (%)** | **Ident (%)** |
| **1*** | Periostin | *Clonorchis sinensis* (358341487) | G | ECPE_0000152101-mRNA-1 | 11.54 | 2 | 5*10^-36^ | 131 | 100 | 72 |
|  |  |  | T | UN02413 | 5.56 | 3 | 2*10^-75^ | 244 | 70 | 59 |
|  | Fasciclin 1-like | *Paragonimus westermani* (119712173) | G | ECPE_0000152101-mRNA-1 | 11.54 | 2 | 5*10^-36^ | 130 | 100 | 76 |
|  |  |  | T | UN02413 | 5.56 | 3 | 1*10^-73^ | 236 | 70 | 57 |
|  | Gynecophoral canal protein | *Schistosoma mansoni* (1354127) | G | ECPE_0000152101-mRNA-1 | 11.54 | 2 | 2*10^-30^ | 113 | 100 | 64 |
|  |  |  | T | UN02413 | 5.56 | 3 | 2*10^-64^ | 211 | 70 | 48 |
| **2** | Leucine aminopeptidase | *P. westermani* (151935446) | G | ECPE_0001158301-mRNA-1 | 59.10 | 14 | 9*10^-68^ | 247 | 92 | 60 |
|  |  |  | T | UN02558 | 9.63 | 3 | 4*10^-45^ | 277 | 43 | 70 |
| **3** | Retinal dehydrogenase 1 | *C. sinensis* (358342257) | G | ECPE_0000537701-mRNA-1 | 29.70 | 20 | 0.0 | 638 | 99 | 71 |
|  |  |  | T | UN10173 | 68.00 | 19 | 0.0 | 634 | 93 | 68 |
| **4** | Hexokinase | *S. haematobium* (8448763000) | G | ECPE_0001750901-mRNA-1 | 58.14 | 16 | 4*10^-118^ | 348 | 100 | 73 |
|  |  |  | T | UN09243 | 18.17 | 18 | 1*10^-159^ | 474 | 41 | 73 |
| **5** | 6-phosphogluconate dehydrogenase | *S. haematobium* (844860554) | G | ECPE_0001241501-mRNA-1 | 42.98 | 17 | 7*10^-122^ | 360 | 90 | 77 |
|  |  |  | T | UN27787 | 29.45 | 8 | 2*10^-65^ | 201 | 84 | 77 |
| **6** | Dihydrolipoamide dehydrogenase | *S. japonicum* (226486712) | G | Unidentified | --- | --- | --- | --- | --- | --- |
|  |  |  | T | UN10835 | 56.00 | 27 | 0.0 | 711 | 71 | 69 |
| **7** | Malate dehydrogenase | *C. sinensis* (358332642) | G | Unidentified | --- | --- | --- | --- | --- | --- |
|  |  |  | T | UN07874 | 44.26 | 27 | 1*10^-55^ | 192 | 46 | 68 |
| **8** | Enolase | *Echinostoma caproni* (112950027) | G | ECPE_0000965901-mRNA-1 | 62.22 | 71 | 0.0 | 892 | 79 | 99 |
|  |  |  | T | UN08624 | 25.45 | 23 | 3*10^-89^ | 283 | 32 | 93 |
| **9** | Putative actin | *S. mansoni* (353233111) | G | ECPE_0000629401-mRNA-1 | 44.41 | 32 | 0.0 | 770 | 100 | 97 |
|  |  |  | T | UN4918 | 39.41 | 13 | 3*10^-95^ | 285 | 85 | 93 |
| **10** | Aldo-keto reductase | *S. japonicum* (2264724280) | G | ECPE_0000877001-mRNA-1 | 11.11 | 4 | 1*10^-75^ | 235 | 81 | 58 |
|  |  |  | T | UN07212 | 10.20 | 4 | 9*10^-71^ | 224 | 85 | 52 |
| **11** | Stress-induced phosphoprotein 1 | *C. sinensis* (350002666) | G | ECPE_0001152901-mRNA-1 | 34.48 | 12 | 0.0 | 546 | 100 | 83 |
|  |  |  | T | UN10046 | 41.00 | 9 | 4*10^-113^ | 598 | 67 | 83 |
| **12** | Aldo-keto reductase | *S. japonicum* (2264724280) | G | ECPE_0000877001-mRNA-1 | 20.09 | 12 | 1*10^-75^ | 235 | 81 | 58 |
|  |  |  | T | UN07212 | 14.20 | 9 | 9*10^-71^ | 224 | 85 | 52 |
| **13** | Aldo-keto reductase | *S. japonicum* (2264724280) | G | ECPE_0000877001-mRNA-1 | 34.19 | 21 | 1*10^-75^ | 235 | 81 | 58 |
|  |  |  | T | UN0516 | 22.37 | 16 | 5*10^-59^ | 192 | 64 | 66 |
| **14** | Putative TyrA protein | *S. japonicum* (226479962) | G | ECPE_0001221701-mRNA-1 | 13.30 | 2 | 4*10^79^ | 254 | 96 | 55 |
|  |  |  | T | UN12149 | 8.56 | 2 | 3*10^-41^ | 201 | 36 | 64 |
| **15** | Cathepsin L | *Fasciola gigantica* (7271891) | G | ECPE_0000839201-mRNA-1 | 17.10 | 4 | 2*10^-149^ | 473 | 92 | 63 |
|  |  |  | T | UN11134 | 11.17 | 3 | 3*10^-144^ | 417 | 83 | 63 |
| **16** | Hydroxyacylglutathione hydrolase | *C. sinensis* (358335388) | G | ECPE_0000120701-mRNA-1 | 72.66 | 12 | 2*10^-45^ | 160 | 76 | 70 |
|  |  |  | T | UN00614 | 8.16 | 4 | 2*10^-31^ | 116 | 57 | 59 |
| **17** | Thiopurine S-methyltransferase | *S. japonicum* (226484786) | G | ECPE_0001499201-mRNA-1 | 26.40 | 6 | 2*10^-59^ | 197 | 98 | 48 |
|  |  |  | T | UN10773 | 19.63 | 8 | 9*10^-83^ | 254 | 69 | 48 |
| **18** | Regulator of microtubule dynamics protein 1 | *S. haematobium* (844834702) | G | ECPE_0000860501-mRNA-1 | 65.76 | 16 | 1*10^-20^ | 95 | 84 | 30 |
|  |  |  | T | UN11076 | 43.15 | 15 | 4*10^-39^ | 140 | 64 | 39 |
| **19** | Glutathione S-transferase | *F. hepatica* (3913799) | G | ECPE_0001537101-mRNA-1 | 9.55 | 2 | 2*10^-106^ | 310 | 100 | 69 |
|  |  |  | T | UN05720 | 8.11 | 2 | 4*10*^-80^ | 245 | 67 | 67 |

^a^ Spot reference number.

^b^ GI accession number in the Protein database of NCBI.

^c^ Database: genome (G) and transcriptome (T).

^d^ Accession number in the *Echinostoma caproni* genome and transcriptome databases.

^e^ Percentage of sequence coverage.

^f^ Number of validated peptides.

^g^ Details of protein blast of translated genome and transcriptome annotations against the NCBInr protein database.

* Three different proteins were associated with the same accession number both in the *E. caproni* genome and transcriptome databases.
