# Supplementary material for: Definitive host influences the proteomic profile of excretory/secretory products of the trematode Echinostoma caproni
Source: Parasit Vectors. 2016 Mar 31;9:185. doi: 10.1186/s13071-016-1465-x (PMC4815245; doi:10.1186/s13071-016-1465-x)
Supplement: Additional file 4: — Biological process-based classification of proteins overexpressed in mice. Significantly overexpressed proteins in the excretory/secretory products of Echinostoma caproni adults obtained from mice, classified according to their Gene Ontology (GO)-predicted biological process. Pie chart represents the number of proteins assigned to each GO category (biological process, level 4). Proteins included in each category are listed below the graph. (PPTX 118 kb) [file 13071_2016_1465_MOESM4_ESM.pptx]

## Slide 1
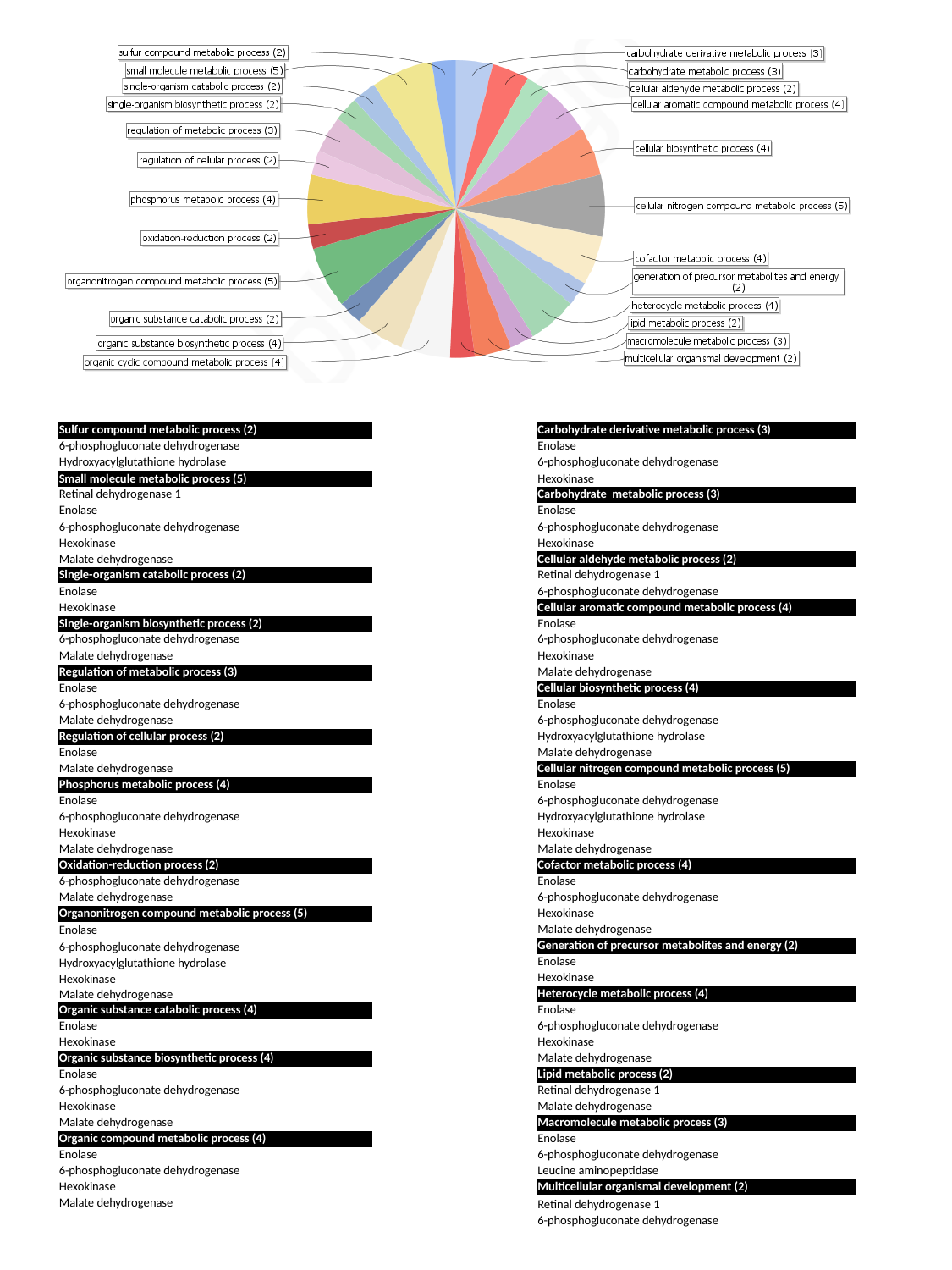

| Sulfur compound metabolic process (2) | | Carbohydrate derivative metabolic process (3) |
| --- | --- | --- |
| 6-phosphogluconate dehydrogenase | | Enolase |
| Hydroxyacylglutathione hydrolase | | 6-phosphogluconate dehydrogenase |
| Small molecule metabolic process (5) | | Hexokinase |
| Retinal dehydrogenase 1 | | Carbohydrate metabolic process (3) |
| Enolase | | Enolase |
| 6-phosphogluconate dehydrogenase | | 6-phosphogluconate dehydrogenase |
| Hexokinase | | Hexokinase |
| Malate dehydrogenase | | Cellular aldehyde metabolic process (2) |
| Single-organism catabolic process (2) | | Retinal dehydrogenase 1 |
| Enolase | | 6-phosphogluconate dehydrogenase |
| Hexokinase | | Cellular aromatic compound metabolic process (4) |
| Single-organism biosynthetic process (2) | | Enolase |
| 6-phosphogluconate dehydrogenase | | 6-phosphogluconate dehydrogenase |
| Malate dehydrogenase | | Hexokinase |
| Regulation of metabolic process (3) | | Malate dehydrogenase |
| Enolase | | Cellular biosynthetic process (4) |
| 6-phosphogluconate dehydrogenase | | Enolase |
| Malate dehydrogenase | | 6-phosphogluconate dehydrogenase |
| Regulation of cellular process (2) | | Hydroxyacylglutathione hydrolase |
| Enolase | | Malate dehydrogenase |
| Malate dehydrogenase | | Cellular nitrogen compound metabolic process (5) |
| Phosphorus metabolic process (4) | | Enolase |
| Enolase | | 6-phosphogluconate dehydrogenase |
| 6-phosphogluconate dehydrogenase | | Hydroxyacylglutathione hydrolase |
| Hexokinase | | Hexokinase |
| Malate dehydrogenase | | Malate dehydrogenase |
| Oxidation-reduction process (2) | | Cofactor metabolic process (4) |
| 6-phosphogluconate dehydrogenase | | Enolase |
| Malate dehydrogenase | | 6-phosphogluconate dehydrogenase |
| Organonitrogen compound metabolic process (5) | | Hexokinase |
| Enolase | | Malate dehydrogenase |
| 6-phosphogluconate dehydrogenase | | Generation of precursor metabolites and energy (2) |
| Hydroxyacylglutathione hydrolase | | Enolase |
| Hexokinase | | Hexokinase |
| Malate dehydrogenase | | Heterocycle metabolic process (4) |
| Organic substance catabolic process (4) | | Enolase |
| Enolase | | 6-phosphogluconate dehydrogenase |
| Hexokinase | | Hexokinase |
| Organic substance biosynthetic process (4) | | Malate dehydrogenase |
| Enolase | | Lipid metabolic process (2) |
| 6-phosphogluconate dehydrogenase | | Retinal dehydrogenase 1 |
| Hexokinase | | Malate dehydrogenase |
| Malate dehydrogenase | | Macromolecule metabolic process (3) |
| Organic compound metabolic process (4) | | Enolase |
| Enolase | | 6-phosphogluconate dehydrogenase |
| 6-phosphogluconate dehydrogenase | | Leucine aminopeptidase |
| Hexokinase | | Multicellular organismal development (2) |
| Malate dehydrogenase | | Retinal dehydrogenase 1 |
| | | 6-phosphogluconate dehydrogenase |
