# Supplementary material for: Definitive host influences the proteomic profile of excretory/secretory products of the trematode Echinostoma caproni
Source: Parasit Vectors. 2016 Mar 31;9:185. doi: 10.1186/s13071-016-1465-x (PMC4815245; doi:10.1186/s13071-016-1465-x)
Supplement: Additional file 5: — Molecular function-based classification of proteins overexpressed in mice. Significantly overexpressed proteins in the excretory/secretory products of Echinostoma caproni adults obtained from mice, classified according to their Gene Ontology (GO)-predicted molecular function. Pie chart represents the number of proteins assigned to each GO category (molecular function, level 3). Proteins included in each category are listed below the graph. (PPTX 77 kb) [file 13071_2016_1465_MOESM5_ESM.pptx]

## Slide 1
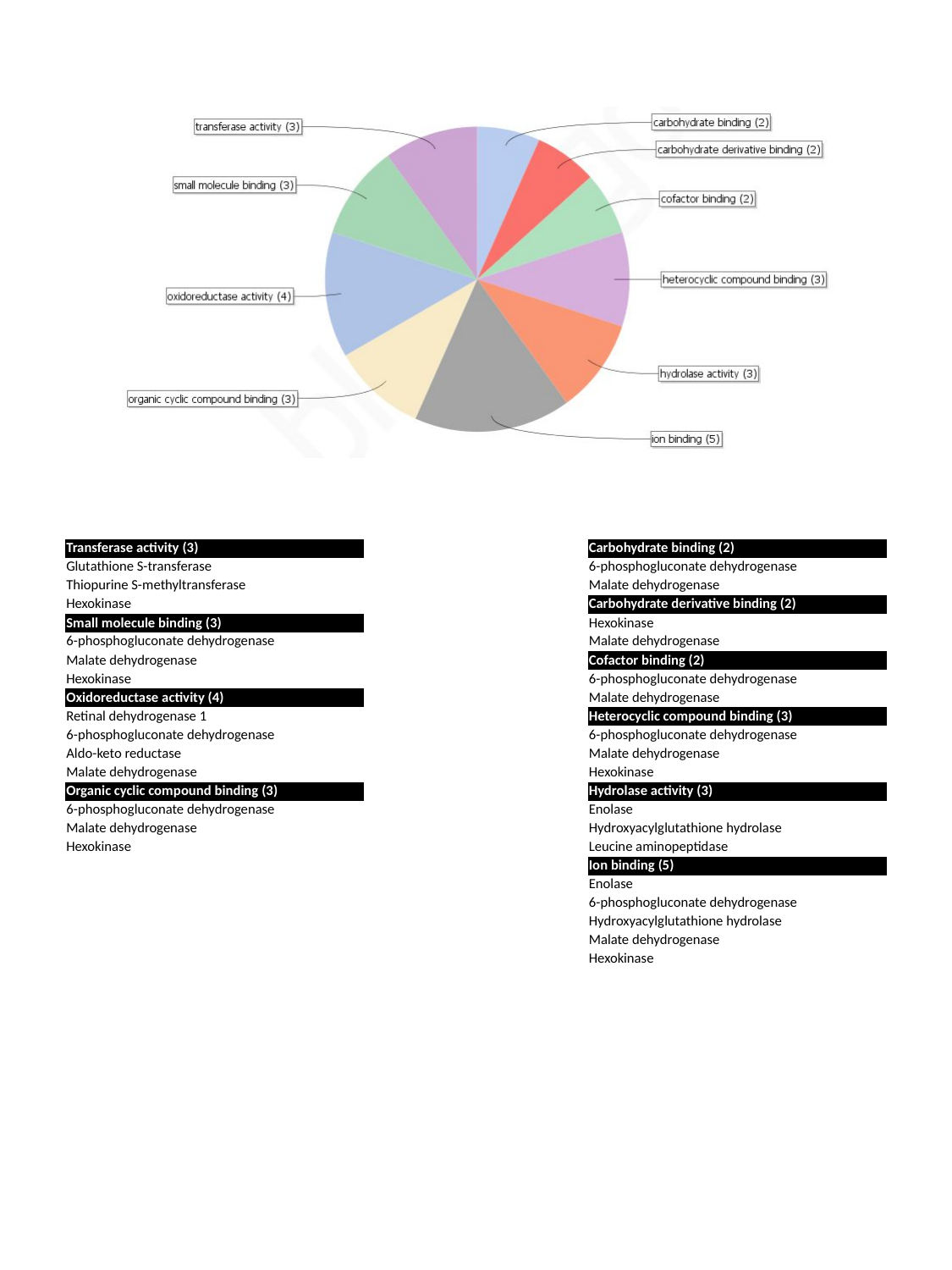

| Transferase activity (3) | | Carbohydrate binding (2) |
| --- | --- | --- |
| Glutathione S-transferase | | 6-phosphogluconate dehydrogenase |
| Thiopurine S-methyltransferase | | Malate dehydrogenase |
| Hexokinase | | Carbohydrate derivative binding (2) |
| Small molecule binding (3) | | Hexokinase |
| 6-phosphogluconate dehydrogenase | | Malate dehydrogenase |
| Malate dehydrogenase | | Cofactor binding (2) |
| Hexokinase | | 6-phosphogluconate dehydrogenase |
| Oxidoreductase activity (4) | | Malate dehydrogenase |
| Retinal dehydrogenase 1 | | Heterocyclic compound binding (3) |
| 6-phosphogluconate dehydrogenase | | 6-phosphogluconate dehydrogenase |
| Aldo-keto reductase | | Malate dehydrogenase |
| Malate dehydrogenase | | Hexokinase |
| Organic cyclic compound binding (3) | | Hydrolase activity (3) |
| 6-phosphogluconate dehydrogenase | | Enolase |
| Malate dehydrogenase | | Hydroxyacylglutathione hydrolase |
| Hexokinase | | Leucine aminopeptidase |
| | | Ion binding (5) |
| | | Enolase |
| | | 6-phosphogluconate dehydrogenase |
| | | Hydroxyacylglutathione hydrolase |
| | | Malate dehydrogenase |
| | | Hexokinase |
